# Establishing Monoclonal Gammopathy of Undetermined Significance as an Independent Pre-Disease State of Multiple Myeloma Using Raman Spectroscopy, Dynamical Network Biomarker Theory, and Energy Landscape Analysis

**DOI:** 10.3390/ijms25031570

**Published:** 2024-01-26

**Authors:** Shota Yonezawa, Takayuki Haruki, Keiichi Koizumi, Akinori Taketani, Yusuke Oshima, Makito Oku, Akinori Wada, Tsutomu Sato, Naoki Masuda, Jun Tahara, Noritaka Fujisawa, Shota Koshiyama, Makoto Kadowaki, Isao Kitajima, Shigeru Saito

**Affiliations:** 1 Research Center for Pre-Disease Science, University of Toyama, Toyama 930-8555, Japan; 2 Graduate School of Science and Engineering, University of Toyama, Toyama 930-8555, Japan; 3 Faculty of Sustainable Design, University of Toyama, Toyama 930-8555, Japan; 4 Division of Presymptomatic Disease, Institute of Natural Medicine, University of Toyama, Toyama 930-0194, Japan; 5 Faculty of Engineering, University of Toyama, Toyama 930-8555, Japan; 6 Faculty of Medicine, University of Toyama, Toyama 930-0194, Japan; 7 Department of Mathematics, State University of New York at Buffalo, Buffalo, NY 14260-2900, USA; 8 Institute for Artificial Intelligence and Data Science, State University of New York at Buffalo, Buffalo, NY 14260-2200, USA

**Keywords:** dynamical network biomarker theory, energy landscape analysis, monoclonal gammopathy of undetermined significance, multiple myeloma, Raman spectroscopy

## Abstract

Multiple myeloma (MM) is a cancer of plasma cells. Normal (NL) cells are considered to pass through a precancerous state, such as monoclonal gammopathy of undetermined significance (MGUS), before transitioning to MM. In the present study, we acquired Raman spectra at three stages—834 NL, 711 MGUS, and 970 MM spectra—and applied the dynamical network biomarker (DNB) theory to these spectra. The DNB analysis identified MGUS as the unstable pre-disease state of MM and extracted Raman shifts at 1149 and 1527–1530 ${\mathrm{cm}}^{-1}$ as DNB variables. The distribution of DNB scores for each patient showed a significant difference between the mean values for MGUS and MM patients. Furthermore, an energy landscape (EL) analysis showed that the NL and MM stages were likely to become stable states. Raman spectroscopy, the DNB theory, and, complementarily, the EL analysis will be applicable to the identification of the pre-disease state in clinical samples.

## 1. Introduction

Plasma cells are blood cells responsible for antibody production and are maintained in a quiescent state in the bone marrow in a normal state (NL). The malignant transformation of plasma cells corresponds to the disease state called multiple myeloma (MM). In this case, plasma cells proliferate in the bone marrow, producing monoclonal antibodies (M proteins) and causing the characteristic symptoms of MM, such as calcium elevation, renal dysfunction, anemia, and bone disease (CRAB criteria). To diagnose MM, the ratio of plasma cells to bone marrow cells must be more than 10%. The primary treatment for MM is chemotherapy, with immunotherapy [1] and targeted therapy [2] also now being applied; however, achieving a cure remains challenging. In contrast, monoclonal gammopathy of undetermined significance (MGUS) is a condition that does not meet the diagnostic criteria for MM because there are fewer than 10% plasma cells in the bone marrow, even though it is clear that these cells are clonally proliferating from the appearance of the M protein [3]. MGUS criteria also include a “serum monoclonal protein <30 g/L” and the “absence of end-organ damage, such as hypercalcemia, renal insufficiency, anemia, and bone lesions or amyloidosis attributed to a plasma cell proliferative disorder”. This condition is the precancerous stage of MM [4]. In the MGUS stage, no characteristic symptoms of MM are present, and patients are followed up without treatment. Approximately 5.3% of individuals older than 70 years old have MGUS [5], and about 1% of MGUS progresses to MM each year [6]. Therefore, a more detailed understanding of the nature of plasma cells in the pre-disease MGUS stage is clinically essential. The boundary that quantitatively distinguishes MM from MGUS is conventionally defined as 10% plasma cells in the bone marrow. Difficulties are associated with identifying differences in plasma cells between MGUS and MM through microscopic observations or analyses of surface markers.

Raman spectroscopy is a label-free and non-invasive analytical method and is one of the vibrational spectroscopies providing information on the molecular composition, molecular structure, and chemical bonding in a sample [7,8,9,10]. Raman microscopic approaches for living cells have been established in combination with multivariate analytical methods such as principal component, classical least-squares, and partial least-squares analyses [11,12,13]. Regarding its preclinical application to hematology, the use of Raman spectroscopy to identify leukocyte subtypes and their activation state induced by pathogen infection was reported by Pistiki et al. [14]. Raman spectroscopy reliably identifies the specific state of each cell, for example, whether it is activated or not and differentiated or not. Predictions of the risk of transition to disease using Raman spectroscopy with the conventional analytical approaches mentioned above remain challenging.

A pre-disease state has historically and qualitatively been defined as an intermediate state between healthy and disease states. The quantitative definition of the pre-disease state has been quite challenging from a mathematical viewpoint. However, Chen et al. constructed the dynamical network biomarker (DNB) theory by introducing the bifurcation theory to the critical transition state before transitioning to the disease state [15]. The DNB theory [15] detects the early warning signals [16,17,18,19,20,21,22,23] of state transitions, specifically the unstable states, in complex networks of biological systems. This theory has been applied to the gene expression profiles of diseases such as lung injury [15,24,25,26], liver cancer [15,24,25,27], breast cancer [25,26,28], influenza infection [26], diabetes [29,30], and metabolic syndrome [31], revealing tipping points for various pre-disease states (unstable states). Moreover, the single sample-based landscape DNB theory has been developed and applied to data on influenza, some cancers [32], coronary atherosclerosis [33], and skin photodamage [34]. Raman spectroscopy has been proposed to prevent invasive or destructive tests, such as the extraction of organ samples, to obtain gene expression profiles. Haruki et al. applied the DNB theory to the Raman spectra of the mouse T-cell activation process, in which naïve cells become fully activated, in non-clinical research [35].

The concept of the energy landscape (EL) has been widely used to describe the existence of multiple stable states in a system and the transitions between them. Recent studies have also estimated ELs from multidimensional data in neuroscience [36,37,38] and ecology [39,40]. Estimated ELs can provide a number of valuable insights into the target system, for example, the number of stable states, the exact positions of local minima, their relative stability, energy barriers between stable states, and the network structure of possible transitions between stable states.

The present study is the first to identify MGUS as the pre-disease state in MM progression by applying the DNB theory to the Raman spectra of three clinically categorized stages: NL, MGUS, and MM. The results of the EL analysis support the identification of the pre-disease state.

## 2. Results

### 2.1. Overview of Raman Spectra

**Figure 1:**
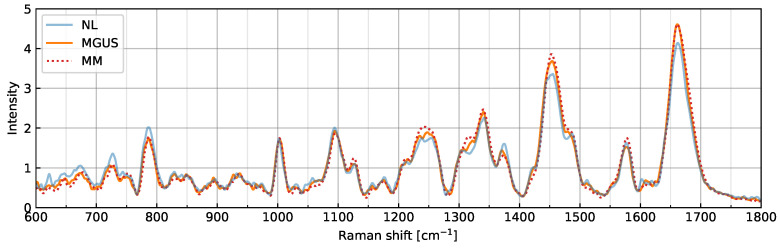
Raman spectral intensities averaged across cells and measurements at NL, MGUS, and MM stages. Each profile is plotted using different colors and line styles.

Figure 1 shows the Raman spectral intensities averaged across cells and measurements at each stage. Preprocessing, such as baseline corrections and smoothing, was effective. Therefore, the profiles of the NL, MGUS, and MM stages were compared, and characteristic peaks at specific Raman shifts were distinguished. For example, in the vicinity of the Raman shift at 1003 ${\mathrm{cm}}^{-1}$, the well-known vibration mode of the phenylalanine ring breathing appeared in all stages.

### 2.2. The Pre-Disease State Identified through the DNB Analysis

**Figure 2:**
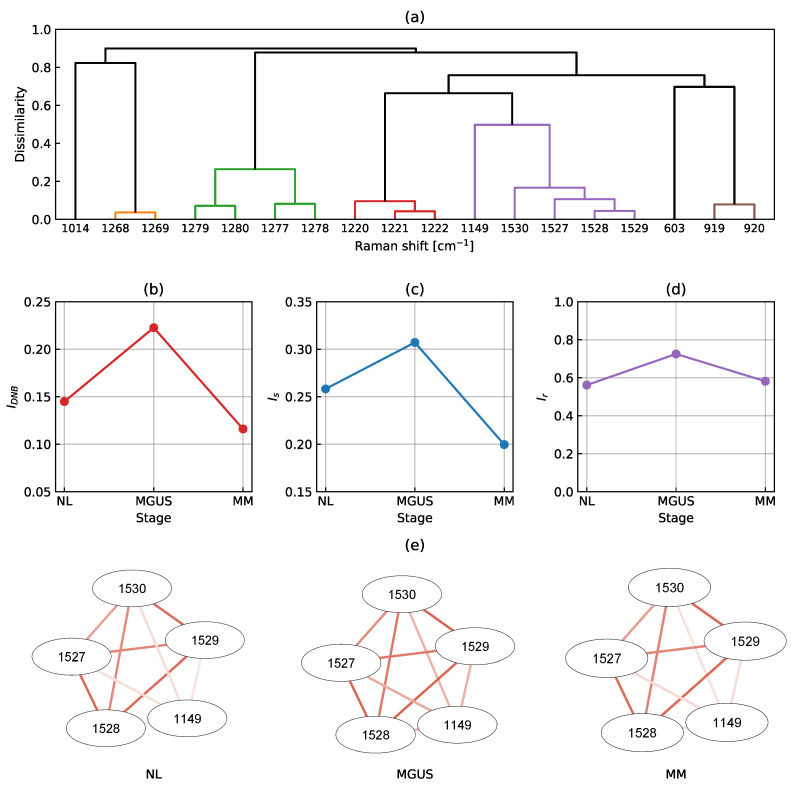
Numerical results obtained through the DNB analysis: (**a**) A dendrogram produced by the hierarchical clustering of 18 significantly fluctuating Raman shifts. Line plots of (**b**) the DNB score, (**c**) average standard deviation, and (**d**) average correlation strength in the largest cluster (the purple group in (**a**)). (**e**) The weighted correlation network. Positive correlation intensities are shown in red.

Figure 2 shows the numerical results obtained through the DNB analysis. An F-test between the control group NL and the experimental group MGUS identified 18 significantly fluctuating Raman shifts. The clustering of these shifts resulted in several clusters, as shown in Figure 2a. The largest cluster, represented in purple, consisted of five Raman shifts, including 1149 and 1527–1530 ${\mathrm{cm}}^{-1}$, which became DNB candidates. Consecutive Raman shifts are denoted with hyphens for simplification. Similarly, the second and third most significant clusters are shown in green (1277–1280 ${\mathrm{cm}}^{-1}$) and red (1220–1222 ${\mathrm{cm}}^{-1}$). However, we did not consider these clusters to be DNB Raman shifts because consecutive Raman shifts may be false correlations.

Figure 2b–d show the line plots of the DNB score, average standard deviation, and average correlation strength, respectively, among the DNB candidates. Typically, the horizontal axis represents time; however, in the present study, we assumed a hypothetical axis sequentially arranging the categorized stages: NL, MGUS, and MM. The DNB score peaked in the MGUS stage, thereby identifying this stage as the pre-disease state (see Figure 2b). Similar results were obtained for the average standard deviation and average correlation strength, as shown in Figure 2c,d. Therefore, we identified Raman shifts at 1149 and 1527–1530 ${\mathrm{cm}}^{-1}$ as DNB Raman shifts. Figure 2e shows the weighted network with the DNB Raman shifts as nodes and their correlation coefficients as edges. Positive correlation intensities are shown in red. Only positive correlations connected to strongly related nodes in the network (there were no negative correlations in this case). Raman shifts at 1527–1530 ${\mathrm{cm}}^{-1}$ strongly correlated with each other at all stages. The correlation of the Raman shift at 1149 ${\mathrm{cm}}^{-1}$ to the set at 1527–1530 ${\mathrm{cm}}^{-1}$ was weak in NL, became strong in MGUS, and was weak in MM. Since these shifts were evaluated from fluctuations and correlations with each other, the graph of the average Raman spectral intensities shows no change around the relevant shifts (see Figure 1).

**Figure 3:**
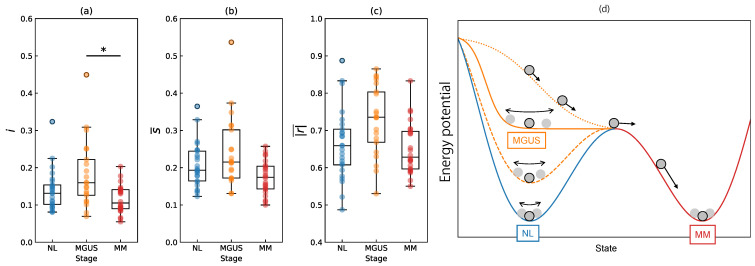
Distributions of (**a**) the DNB score *i*, (**b**) average standard deviation $\bar{s}$, and (**c**) average correlation strength $\bar{\left|r\right|}$ of each patient with box-and-whisker plots. All values were averaged among the Raman spectra in approximately 15–20 cells derived from each patient. An asterisk (*) denotes significance: * p<0.005 in (**a**). Each dot corresponds to the respective patient data. (**d**) A conceptual diagram of the energy potential [15,35]. The potentials of NL, MGUS, and MM are plotted using different colors. The orange dotted line shows the potential of transitioning to the MM stage. The orange dashed line shows the potential close to the NL stage.

Figure 3 shows the distributions of the DNB score *i* (Figure 3a), the average standard deviation $\bar{s}$ (Figure 3b), and the average correlation strength $\bar{\left|r\right|}$ (Figure 3c) of each patient with box-and-whisker plots. Additionally, Figure 3d shows a conceptual diagram of the energy potential. The score *i* satisfied the product of two quantities, $\bar{s}\times \bar{\left|r\right|}$. The results of two numerical analyses, the DNB and EL analyses, indicated stable states for NL and MM and an unstable state for MGUS. As shown in Figure 3a,b, the dots for MGUS are more widely distributed than those of NL and MM. The correlation at the MGUS stage is higher than that at the NL and MM stages (see Figure 3c). The box plots for MGUS are positioned higher than those of the other stages in all quantities. This result aligns with the findings in Figure 2. Welch’s t-test detected a significant difference in the means of the DNB scores (NL vs. MGUS and MGUS vs. MM). The significant difference in the means of the MGUS and MM stages was p=0.004, which was lower than the Bonferroni-adjusted threshold of 0.05/2=0.025 (see the asterisk in Figure 3a). However, no significant difference was observed between the means of the NL and MGUS stages (p=0.064).

We assume a pseudo-time-series stage in which NL, MGUS, and MM are aligned. As shown in Figure 3d, NL is stable because plasma cells may have normal morphological functions (the blue line). Nevertheless, various factors impairing health can affect this state and increase the potential baseline. In the transitional state of MGUS (the orange line), the shape of the potential flattens or deteriorates, and the system transitions toward MM (the red line). The upper part of the distribution in Figure 3a appears to be transitioning to MM because of the higher DNB score, corresponding to the orange dotted line in Figure 3d. In contrast, the lower part appears to be located on the stable NL side, shown by the orange dashed line (see Figure 3d). Therefore, the DNB Raman shifts obtained from all patients may be able to predict the outcome of each MGUS patient.

### 2.3. Frequent Spectral Patterns Revealed through the EL Analysis

**Figure 4:**
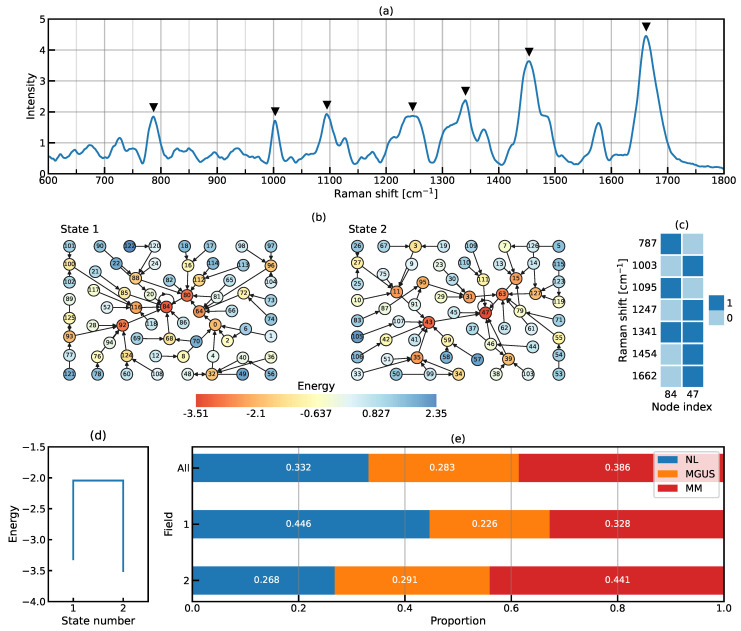
Numerical results obtained through the EL analysis: (**a**) The selection of Raman shifts with the top seven peaks. Inverted triangles indicate the selected variables. (**b**) Basin graphs. (**c**) Activity patterns at the local minimum of each state. Active Raman shifts are colored. (**d**) Disconnectivity graph. (**e**) Proportion of data points at each stage. The total may not precisely be equal to 1 due to rounding to the fourth decimal place.

Figure 4 shows the numerical results obtained through the EL analysis. The average of the entire spectrum, including all stages, is shown in Figure 4a. The variables used for this analysis were the top seven peaks of the spectrum, located at 787, 1003, 1095, 1247, 1341, 1454, and 1662 ${\mathrm{cm}}^{-1}$ (see the inverted triangle markers). Each variable was assigned a value of 1 if it was greater than the average of all samples and 0 otherwise. We refer to the combination of seven binarized power signals as the activity pattern. Figure 4b shows basin graphs of how the different activity patterns were related to each other. The analysis identified two distinct macroscopic states: State 1 and State 2. In State 1, node index 84 was the local minimum of energy or the bottom of an attractive basin, indicated by a red node. The binary expression of 84 was 1010100, which indicated that the intensities of the Raman shifts at 787, 1095, and 1341 ${\mathrm{cm}}^{-1}$ were higher than their average, and vice versa at the four other Raman shifts (see Figure 4c). Similarly, node index 47 (0101111 in binary notation) was the local minimum of State 2 (see Figure 4b,c). Figure 4d shows a disconnectivity graph, indicating that the minimum energies for State 1 and State 2 were −3.32 and −3.51, respectively.

We defined the sets of nodes with energy smaller than −2.05 as fields (see Figure 4d). A field represents a set of activity patterns frequently visited within the given state. Field 1 within State 1 consisted of the node set {64,80,84,92,116}, and Field 2 within State 2 consisted of the node set {11,15,31,35,43,63,95}. Figure 4e shows the proportions of NL, MGUS, and MM data points within each field. In Field 1, data for NL were more abundant compared to MGUS and MM, accounting for 44.6% of the total; therefore, State 1, when characterized by Field 1, appeared to be a normal-stable state. In contrast, MM was overrepresented (accounting for 44.1% of the total) in Field 2, suggesting that State 2, characterized by Field 2, was closer to a disease-stable state.

## 3. Discussion

The Raman spectral data obtained and analyzed in the present study consisted of 1201 variables that reflect molecular dynamics in living plasma cells. In a comparison of the averaged spectral data (see Figure 1), the spectral features of each stage slightly differed. It is insufficient to consider MGUS as a pre-disease state before the transition to malignancy by only focusing on specific Raman shifts. The results of the DNB analysis (see Figure 2 and Figure 3) using Raman spectroscopic data obtained from the different clinical conditions, namely NL, MGUS, and MM, strongly suggest that MGUS is the transition state from the healthy to the disease state during the onset of MM. These three categories were sampled from independent patients but may be regarded as pseudo-time-course states in the biological process of cancer progression. According to the DNB theory, significant fluctuations and strong correlations in Raman intensities represent early warning signals for state transitions [15,35]. Therefore, DNB Raman shifts may be considered potential surrogate markers for the prognosis of MGUS.

Alternatively, EL analysis is a promising tool for understanding state transitions. Its application to Raman spectral data with 1201 dimensions is typically impossible. However, the selection of seven Raman shifts with strong peak intensities from the 1201 shifts enabled us to use EL analysis in the present study. The 2515 Raman spectra of the three stages (NL, MGUS, and MM) were sufficiently large and available for the EL analysis. The average number of Raman spectra per activity pattern was approximately 20, which appeared to be sufficiently large to compute the EL and its derivatives. With advances in Raman spectroscopy, the automatic and efficient acquisition of numerous Raman spectra is becoming a reality. The combined use of Raman spectroscopy and EL analysis is expected to be beneficial for investigations of stable and unstable states in targeted systems.

However, there were several limitations in the research design and analytical approaches used in the present study. The biological implication of DNB Raman shifts remains challenging. Raman peak assignment only provides information on, for example, molecular moieties and chemical bonding. In addition, the correlation between increased DNB scores in MGUS and the risk of progression to MM is still only speculation. Further experiments and analyses, combined with transcriptome and proteome analyses, need to be performed alongside Raman spectroscopic measurements.

The transition from myeloma to plasma cell leukemia was discussed with consideration of genetic evolution, which may occur in multiple myeloma. Under this condition, MM may be regarded as a pre-disease state (tipping point) of plasma cell leukemia. However, this does not contradict the results of the present study, which identified MGUS as the pre-disease state of MM. This is because the complex network indicating tipping points is considered to be different. Multiple tipping points are anticipated during the process of plasma cell transformation.

Since CD138+, a surface maker on plasma cells, is expressed in NL, MGUS, and MM, difficulties are associated with their distinction. A previous study reported that 14q32 rearrangements and chromosome 13 deletions, characteristic of MM, were also present in 46 and 20% of MGUS, respectively, but were absent in NL [41]. Therefore, conventional quantitative diagnostic methods have been used.

In the present study, we proposed an approach to detect qualitative changes in plasma cells from NL to MM using Raman spectroscopy, reconsidering MGUS as a new disease category. This approach differs from quantitative diagnostic methods, distinguishing MGUS and MM based on the percentage of plasma cells in the bone marrow. Our methodology can be further advanced by combining it with conventional flow cytometry techniques. For example, the flow cytometry device can be equipped with another laser to measure Raman scattering. With these improvements, our methodology may lead to a rapid and high-throughput diagnosis. In addition, the combination of not only CD138 but also CD19 and CD56, which are surface antigens that help distinguish the malignancy of plasma cells [42], will provide more information; however, a small fraction of CD56+ polyclonal plasma cells also exists in healthy individuals [43]. As shown in Figure 4, the presence of two basins in NL and MM, but not MGUS, is notable. Although CD56 is an MM marker, plasma cells (CD138+) comprise CD56+ and CD56- cells in the MGUS stage. In the future, we aim to perform an EL analysis of CD138+/CD56+ and CD138+/CD56- cells.

The present study is the first to demonstrate, using objective mathematical indicators, that MGUS is not just an intermediate stage but also an independent pre-disease state with unique characteristics. This type of methodology can be applied to many other diseases preceded by specific conditions that may be recognized as pre-disease states. Therefore, our results will significantly impact various fields.

## 4. Materials and Methods

### 4.1. Overview of our Experimental Design

**Figure 5:**
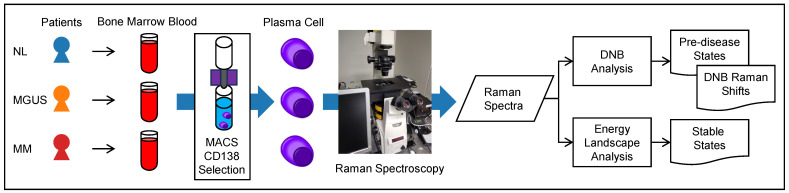
Our experimental design. Upstream Phase: Raman spectra acquisition. Downstream Phase: Numerical analyses.

Figure 5 shows an overview of our experimental design, which is broadly divided into two main phases. In the upstream phase, Raman spectroscopy acquires the Raman spectra from clinical samples. The spectral data of MGUS and MM are the experimental group, and NL is the control group. In the downstream phase, the DNB theory is applied to these spectra (DNB analysis), identifying the pre-disease state, and the EL analysis detects stable states in plasma cell changes.

### 4.2. Clinical Samples

Patients with or suspected of having hematological malignancies and who underwent a bone marrow examination at the Department of Hematology, Toyama University Hospital, between January 2017 and August 2023 were included. The present study was conducted according to the Declaration of Helsinki and was approved by the Ethics Committees of Toyama University Hospital (approval number R2020081). Written informed consent was obtained from all patients before study participation.

In the present study, CD138+ cells were reliably selected from bone marrow cells using magnetic beads equipped with CD138 antibodies [44] in the absence of negative controls [45]. Lymphoprep^TM^ (Serumwerk Bernburg AG, Bernburg, Germany) isolated bone marrow mononuclear cells from bone marrow puncture fluid. CD138+ cells were isolated using CD138 immunomagnetic beads, MACSprep^TM^ Multiple Myeloma CD138 MicroBeads (Order no. 130-111-744) (Miltenyi Biotec, Auburn, CA, USA), according to the manufacturer’s protocol. We selected normal control samples (NL) that showed no infiltration of malignant lymphoma cells in bone marrow samples, which were collected to identify the clinical stage in patients with malignant lymphoma [46,47,48,49].

### 4.3. Instrumentation and Measurement: Utilizing Raman Spectroscopy

The Raman spectral data of NL, MGUS, and MM patients were acquired using a custom-designed Raman microscopy system [50]. In brief, an inverted microscope (ECLIPSE Ti-E, Nikon, Japan) equipped with a motorized stage was used to observe cells with a 60× oil immersion objective lens (CFI Apochromat TIRF 60XC Oil, Nikon, Tokyo, Japan). A DPSS laser (Samba 532 nm, Cobolt, Solna, Sweden) was introduced into the microscope as an excitation source, and its intensity was adjusted between 0.2 and 20 mW at the sample point. Raman scattering was collected at the same objective and returned along the same optical path (RPM-532, Airix, Tokyo, Japan). A spectrometer (SP2150, Teledyne Princeton Instruments, Trenton, NJ, USA) with a CCD (iVac BI-DD, Andor Technology, Belfast, UK) was used to acquire Raman spectral data. The system was designed to measure living plasma cells floating in a chamber (Micro-chamber INT-750, INTROTEC, Kawasaki, Japan), which was sealed at the top and bottom by quartz glass and contained PBS.

### 4.4. Datasets

Table 1 shows a summary of the data counts. Bone marrow blood samples were obtained from 25 NL, 21 MGUS, and 24 MM patients. MACS CD138 selection extracted plasma cells from each sample, and Raman spectroscopy provided their spectra. We acquired 834 NL, 711 MGUS, and 970 MM Raman spectra; therefore, the total count was 2515. The spectra of all samples were subjected to DNB and EL analyses.

### 4.5. Preprocessing

Raman spectra obtained from cells contain significant noise components and, thus, require a pre-treatment for analyses. Raman spectra were converted at equal intervals to facilitate each subsequent process. The subtraction of background intensities caused by water and quartz from the Raman spectra removed their effects. Broad noise components, such as fluorescence, were removed by baseline corrections using a rolling ball algorithm. The rolling ball algorithm used the Python module skimage.restoration.rolling_ball, where the radius was 50 ${\mathrm{cm}}^{-1}$ in the wavenumber range from 600 to 1800 ${\mathrm{cm}}^{-1}$. The moving average of the Savitzky–Golay filter suppressed delicate noise components in the spectra, a process referred to as smoothing. The window length (number of data used for approximation) was seven, and the polynomial degree was three with the Python module scipy.signal.savgol_filter (version 1.11.3). The intensity at each Raman shift was normalized by dividing by the average of all spectra.

### 4.6. DNB Analysis

In many studies on the DNB theory, health and disease states are characterized as stable, whereas the pre-disease state (also called the transition state or tipping point) is reported to be unstable. We introduced this logic into the model of MM progression, assuming that NL and MM, which are the start and end points, respectively, are in stable states. We also hypothesized that MGUS, an intermediate stage in the progression from NL to MM, is unstable.

We related NL and MM to the bottom of the potential, corresponding to a locally stable state. We also related MGUS to the flat region of the potential, corresponding to the transition state. In this case, the state may move significantly from side to side over the potential, resulting in large fluctuations. This phenomenon implies increased fluctuations in the distribution and/or density of intracellular molecules, corresponding to the observed Raman shifts. In other words, some cells contain more molecules, whereas others contain fewer molecules.

The present study utilized the DNB analysis based on the study by Haruki et al. [35]. Raman shifts in our dataset ranged between 600 and 1800 ${\mathrm{cm}}^{-1}$ in increments of 1; therefore, we handled 1201 variables. The DNB analysis involves (1) using an F-test to evaluate fluctuations in each Raman shift, (2) employing hierarchical clustering with the correlation between these shifts to define DNB candidates, and (3) identifying the pre-disease state and DNB variables based on the peaks of DNB scores.

Data for each stage are described as the matrix $X=x_{ik}$ (i=1,2,…,N and k=1,2,…,K), where *i* and *k* are the indices of the variables (Raman shifts) and a sample of the Raman spectrum, respectively; *N* is the number of Raman shifts; and *K* is the number of samples at that stage. The mean $m_{i}\left(X\right)$ and sample standard deviation $s_{i}\left(X\right)$ are defined as

(1) $$\begin{array}{ccc} m_{i}\left(X\right) & = & \frac{\sum_{k=1}^{K}x_{ik}}{K}, \end{array}$$

(2) $$\begin{array}{ccc} s_{i}\left(X\right) & = & \sqrt{\frac{\sum_{k=1}^{K}{\left(x_{ik}-m_{i}\left(X\right)\right)}^{2}}{K-1}}, \end{array}$$

where a large $s_{i}\left(X\right)$ corresponds to a large fluctuation.

The one-tailed F-test evaluated the intensity of fluctuations in each variable. The null hypothesis was that the variance in two groups (MGUS or MM as the experimental group and NL as the control group) was equal. The rejection of this null hypothesis suggested that the two groups exhibited unequal variance, indicating significantly fluctuating variables. Multiple testing corrections using the Benjamini–Hochberg method then suppressed the false discovery rate, converting the *p*-value obtained from the F-test to the *q*-value. Variables were considered significantly fluctuating if q<0.05.

Pearson’s correlation coefficient is defined as follows:

(3) $$\begin{array}{ccc} r_{ij}\left(X\right) & = & \frac{\sum_{k=1}^{K}\left(x_{ik}-m_{i}\left(X\right)\right)(x_{jk}-m_{j}\left(X\right))}{\left(K-1\right)\;s_{i}\left(X\right)s_{j}\left(X\right)}, \end{array}$$

where i,j=1,2,…,N. A high correlation coefficient indicates that the spectral intensities of the *i*th and *j*th Raman shifts co-fluctuated across samples.

The coefficient $r_{ij}$ between significantly fluctuating Raman shifts was subsequently used to define dissimilarity as $d=1-|r_{ij}|$ for hierarchical clustering, resulting in a dendrogram. The dissimilarity cut-off in the dendrogram was set at 0.5, leading to several clusters being identified as DNB candidates. DNB candidates were selected based on cluster size (the number of included Raman shifts): the largest clusters and those more than half the size of the largest clusters. We then computed the DNB score $I_{DNB}$, which is the product of the average standard deviation $I_{s}$, and the average correlation strength $I_{r}$ of Raman shifts for each DNB candidate. The peak of the DNB score indicated the critical transition state in time-series data. However, based on clinical insights, we sequentially arranged the three stages—NL, MGUS, and MM—for categorized data in the present study. The peak at the transition state indicated that the DNB candidate became the DNB Raman shifts.

We also attempted to provide feedback on the results of the DNB analysis with the entire dataset for each patient. Similar to the procedure in the DNB analysis, the DNB score for each patient was individually calculated in an extra phase and has the potential to serve as an indicator of subsequent transitions to different stable states.

### 4.7. EL Analysis

Each binary vector, composed of a combination of 1 (i.e., active) or 0 (i.e., inactive) across different Raman shifts, defined an activity pattern. By definition, an activity pattern with a high frequency of occurrence was considered to have low energy, and vice versa. Activity patterns with low energy values were interpreted as being relatively stable.

We obtained a total of 2515 data points. We set the number of variables to seven, which implied $2^{7}=128$ binary activity patterns and 2515/128≈19.6 data points per activity pattern on average. According to the guidelines provided by a previous study [37], we considered the application of the EL analysis with seven variables to the present dataset to be reasonable. We used seven Raman shifts corresponding to the seven strongest peaks of the Raman spectra for the EL analysis. We integrated the Raman data of patients and cells from the NL, MGUS, and MM stages and regarded the combined dataset as pseudo-time-series data. An underlying implicit assumption was that plasma cells dynamically transition from one pattern to another, which may occur on a slow timescale, and our samples from different patients represented various patterns appearing in the course of these dynamics.

Each of the 2515 filtered data points was converted to a seven-dimensional binarized activity pattern consisting of 0 and 1. For each variable (i.e., Raman shift), values (i.e., spectral intensities) greater than the mean value in the 2515 samples were set to 1, and the others to 0. An Ising model (also known as the pairwise maximum entropy model) was fitted to binarized data [37]. The parameters of the Ising model were estimated using the maximum likelihood method. The Ising model fit to binarized data provided the energy of each activity pattern, the local minima of energy, their basins, and a disconnectivity graph.

Based on the energy value associated with each of the 128 activity patterns, we constructed a directed network by drawing an arrow (i.e., a directed edge) from a higher-energy activity pattern to a lower-energy activity pattern, where activity patterns defined nodes. By definition, each node has one directed edge that points to the activity pattern with the lowest energy in its neighborhood. The resulting directed network is called a basin graph. The directed path from any *i*th node in the basin graph indicates a unique path toward the most stable activity pattern in the basin to which the *i*th node belongs. The energy barrier is known as the lowest energy uphill that needs to be overcome in order to transition toward different stable states. The disconnectivity graph includes the number and energies of stable states and energy barriers. These analyses were implemented using a Python library, which is available at https://github.com/okumakito/elapy (accessed on 4 August 2023).

## 5. Conclusions

In the present study, we acquired 2515 Raman spectra from NL, MGUS, and MM states through Raman spectroscopy and applied the DNB theory to these spectra. The DNB analysis identified MGUS as the pre-disease state of MM and extracted Raman shifts 1149 and 1527–1530 ${\mathrm{cm}}^{-1}$ as DNB variables. The EL analysis complementarily supported the idea of NL and MM stages being more prevalent in their respective stable states. Therefore, we established MGUS as the independent pre-disease state of MM in clinical samples.

These results will lead to new applicable ideas for predicting disease progression from MGUS to MM. In the DNB theory, MGUS with a high DNB score is regarded as an unstable state that may be more prone to transitioning to MM, which is considered a disease-stable state.

Therefore, future studies should investigate the correlation between the DNB scores of MGUS and the frequency of subsequent transitions to MM. Additionally, further investigations are warranted to elucidate the molecular biological characteristics of MGUS that transition to MM. These characteristics should be analyzed in an integrated manner using Raman spectroscopic, DNB, and EL data to accurately detect early-warning signals for the subsequent transition to MM in the MGUS state, thereby leading to clinical applications.

## Figures and Tables

**Table 1:** Summary of the data counts.

			NL	MGUS	MM	Total
Patients			25	21	24	70
Plasma cells			396	347	471	1214
	max.		21	25	36	
	median	(cells/patient)	15	15	15	
	min.		9	9	5	
Raman spectra			834	711	970	2515
	max.		4	4	4	
	median	(measurements/cell)	2	2	2	
	min.		2	1	2	

## Data Availability

The data presented in this study are not available due to privacy and ethical reasons.

## Abbreviations

The following abbreviations are used in this manuscript:

DNB	Dynamical Network Biomarker
EL	Energy Landscape
MGUS	Monoclonal Gammopathy of Undetermined Significance
MM	Multiple Myeloma
NL	Normal

## References

[1] Abramson H.N. (2023). Immunotherapy of Multiple Myeloma: Current Status as Prologue to the Future. Int. J. Mol. Sci..

[2] Leow C.C.-Y., Low M.S.Y. (2021). Targeted Therapies for Multiple Myeloma. J. Pers. Med..

[3] Rajkumar S.V., Dimopoulos M.A., Palumbo A., Blade J., Merlini G., Mateos M.V., Kumar S., Hillengass J., Kastritis E., Richardson P. (2014). International Myeloma Working Group updated criteria for the diagnosis of multiple myeloma. Lancet Oncol..

[4] Kumar S.K., Rajkumar S.V. (2018). The multiple myelomas—Current concepts in cytogenetic classification and therapy. Nat. Rev. Clin. Oncol..

[5] Kyle R.A., Therneau T.M., Rajkumar S.V., Larson D.R., Plevak M.F., Offord J.R., Dispenzieri A., Katzmann J.A., Melton L.J. (2006). Prevalence of Monoclonal Gammopathy of Undetermined Significance. N. Engl. J. Med..

[6] Kyle R.A., Therneau T.M., Rajkumar S.V., Offord J.R., Larson D.R., Plevak M.F., Melton L.J. (2002). A Long-Term Study of Prognosis in Monoclonal Gammopathy of Undetermined Significance. N. Engl. J. Med..

[7] Movasaghi Z., Rehman S., Rehman I.U. (2007). Raman Spectroscopy of Biological Tissues. Appl. Spectrosc. Rev..

[8] Krafft C., Schmitt M., Schie I.W., Cialla-May D., Matthäus C., Bocklitz T., Popp J. (2017). Label-Free Molecular Imaging of Biological Cells and Tissues by Linear and Nonlinear Raman Spectroscopic Approaches. Angew. Chem..

[9] Dodo K., Fujita K., Sodeoka M. (2022). Raman Spectroscopy for Chemical Biology Research. J. Am. Chem. Soc..

[10] Watanabe T.M., Sasaki K., Fujita H. (2022). Recent Advances in Raman Spectral Imaging in Cell Diagnosis and Gene Expression Prediction. Genes.

[11] Notingher I., Jell G., Notingher P.L., Bisson I., Tsigkou O., Polak J.M., Stevens M.M., Hench L.L. (2005). Multivariate analysis of Raman spectra for in vitro non-invasive studies of living cells. J. Mol. Struct..

[12] Chan J.W., Taylor D.S., Lane S.M., Zwerdling T., Tuscano J., Huser T. (2008). Nondestructive identification of individual leukemia cells by laser trapping Raman spectroscopy. Anal. Chem..

[13] Ishigaki M., Hitomi H., Ozaki Y., Nishiyama A. (2022). Exposing intracellular molecular changes during the differentiation of human-induced pluripotent stem cells into erythropoietin-producing cells using Raman spectroscopy and imaging. Sci. Rep..

[14] Pistiki A., Ramoji A., Ryabchykov O., Thomas-Rüddel D., Press A.T., Makarewicz O., Giamarellos-Bourboulis E.J., Bauer M., Bocklitz T., Popp J. (2021). Biochemical Analysis of Leukocytes after In Vitro and In Vivo Activation with Bacterial and Fungal Pathogens Using Raman Spectroscopy. Int. J. Mol. Sci..

[15] Chen L., Liu R., Liu Z.P., Li M., Aihara K. (2012). Detecting early-warning signals for sudden deterioration of complex diseases by dynamical network biomarkers. Sci. Rep..

[16] Carpenter S.R., Brock W.A. (2006). Rising variance: A leading indicator of ecological transition. Ecol. Lett..

[17] Dakos V., Scheffer M., Van Nes E.H., Brovkin V., Petoukhov V., Held H. (2008). Slowing down as an early warning signal for abrupt climate change. Proc. Natl. Acad. Sci. USA.

[18] Scheffer M., Bascompte J., Brock W.A., Brovkin V., Carpenter S.R., Dakos V., Held H., Van Nes E.H., Rietkerk M., Sugihara G. (2009). Early-warning signals for critical transitions. Nature.

[19] Moon H., Lu T.C. (2015). Network Catastrophe: Self-Organized Patterns Reveal both the Instability and the Structure of Complex Networks. Sci. Rep..

[20] Veraart A.J., Faassen E.J., Dakos V., Van Nes E.H., Lürling M., Scheffer M. (2012). Recovery rates reflect distance to a tipping point in a living system. Nature.

[21] Dakos V., Carpenter S.R., Brock W.A., Ellison A.M., Guttal V., Ives A.R., Kéfi S., Livina V., Seekell D.A., Van Nes E.H. (2012). Methods for Detecting Early Warnings of Critical Transitions in Time Series Illustrated Using Simulated Ecological Data. PLoS ONE.

[22] Olthof M., Hasselman F., Strunk G., Van Rooij M., Aas B., Helmich M.A., Schiepek G., Lichtwarck-Aschoff A. (2020). Critical Fluctuations as an Early-Warning Signal for Sudden Gains and Losses in Patients Receiving Psychotherapy for Mood Disorders. Clin. Psychol. Sci..

[23] Bury T.M., Sujith R.I., Pavithran I., Scheffer M., Lenton T.M., Anand M., Bauch C.T. (2021). Deep learning for early warning signals of tipping points. Proc. Natl. Acad. Sci. USA.

[24] Liu R., Li M., Liu Z.-P., Wu J., Chen L., Aihara K. (2012). Identifying critical transitions and their leading biomolecular networks in complex diseases. Sci. Rep..

[25] Chen P., Liu R., Li Y., Chen L. (2016). Detecting critical state before phase transition of complex biological systems by hidden Markov model. Bioinformatics.

[26] Liu R., Yu X., Liu X., Xu D., Aihara K., Chen L. (2014). Identifying critical transitions of complex diseases based on a single sample. Bioinformatics.

[27] Yang B., Li M., Tang W., Liu W., Zhang S., Chen L., Xia J. (2018). Dynamic network biomarker indicates pulmonary metastasis at the tipping point of hepatocellular carcinoma. Nat. Commun..

[28] Jiang F., Yang L., Jiao X. (2023). Dynamic network biomarker to determine the critical point of breast cancer stage progression. Breast Cancer.

[29] Liu X., Liu R., Zhao X.-M., Chen L. (2013). Detecting early-warning signals of type 1 diabetes and its leading biomolecular networks by dynamical network biomarkers. BMC Med. Genom..

[30] Li M., Zeng T., Liu R., Chen L. (2014). Detecting tissue-specific early warning signals for complex diseases based on dynamical network biomarkers: Study of type 2 diabetes by cross-tissue analysis. Brief Bioinform..

[31] Koizumi K., Oku M., Hayashi S., Inujima A., Shibahara N., Chen L., Igarashi Y., Tobe K., Saito S., Kadowaki M. (2019). Identifying pre-disease signals before metabolic syndrome in mice by dynamical network biomarkers. Sci. Rep..

[32] Liu X., Chang X., Leng S., Tang H., Aihara K., Chen L. (2019). Detection for disease tipping points by landscape dynamic network biomarkers. Natl. Sci. Rev..

[33] Ge J., Song C., Zhang C., Liu X., Chen J., Dou K., Chen L. (2020). Personalized Early-Warning Signals during Progression of Human Coronary Atherosclerosis by Landscape Dynamic Network Biomarker. Genes.

[34] Zhang C., Zhang H., Ge J., Mi T., Cui X., Tu F., Gu X., Zeng T., Chen L. (2022). Landscape dynamic network biomarker analysis reveals the tipping point of transcriptome reprogramming to prevent skin photodamage. J. Mol. Cell Biol..

[35] Haruki T., Yonezawa S., Koizumi K., Yoshida Y., Watanabe T.M., Fujita H., Oshima Y., Oku M., Taketani A., Yamazaki M. (2022). Application of the Dynamical Network Biomarker Theory to Raman Spectra. Biomolecules.

[36] Watanabe T., Masuda N., Megumi F., Kanai R., Rees G. (2014). Energy landscape and dynamics of brain activity during human bistable perception. Nat. Commun..

[37] Ezaki T., Watanabe T., Ohzeki M., Masuda N. (2017). Energy landscape analysis of neuroimaging data. Philos. Trans. R. Soc. A.

[38] Watanabe T., Rees G. (2017). Brain network dynamics in high-functioning individuals with autism. Nat. Commun..

[39] Suzuki K., Nakaoka S., Fukuda S., Masuya H. (2021). Energy landscape analysis elucidates the multistability of ecological communities across environmental gradients. Ecol. Monogr..

[40] Fujita H., Ushio M., Suzuki K., Abe M.S., Yamamichi M., Iwayama K., Canarini A., Hayashi I., Fukushima K., Fukuda S. (2023). Alternative stable states, nonlinear behavior, and predictability of microbiome dynamics. Microbiome.

[41] Avet-Loiseau H., Facon T., Daviet A., Godon C., Rapp M.J., Harousseau J.L., Grosbois B., Bataille R. (1999). 14q32 translocations and monosomy 13 observed in monoclonal gammopathy of undetermined significance delineate a multistep process for the oncogenesis of multiple myeloma. Intergroupe Francophone du Myélome. Cancer Res..

[42] Harada H., Kawano M.M., Huang N., Harada Y., Iwato K., Tanabe O., Tanaka H., Sakai A., Asaoku H., Kuramoto A. (1993). Phenotypic difference of normal plasma cells from mature myeloma cells. Blood.

[43] Tembhare P.R., Yuan C.M., Venzon D., Braylan R., Korde N., Manasanch E., Zuchlinsky D., Calvo K., Kurlander R., Bhutani M. (2014). Flow cytometric differentiation of abnormal and normal plasma cells in the bone marrow in patients with multiple myeloma and its precursor diseases. Leuk. Res..

[44] Sato T., Tatekoshi A., Takada K., Iyama S., Kamihara Y., Jawaid P., Rehman M.U., Noguchi K., Kondo T., Kajikawa S. (2019). DPP8 is a novel therapeutic target for multiple myeloma. Sci. Rep..

[45] Racanelli V., Leone P., Frassanito M.A., Brunetti C., Perosa F., Ferrone S., Dammacco F. (2010). Alterations in the antigen processing-presenting machinery of transformed plasma cells are associated with reduced recognition by CD8+ T cells and characterize the progression of MGUS to multiple myeloma. Blood.

[46] Ocqueteau M., Orfao A., Almeida J., Bladé J., González M., García-Sanz R., López-Berges C., Moro M.J., Hernández J., Escribano L. (1998). Immunophenotypic characterization of plasma cells from monoclonal gammopathy of undetermined significance patients. Implications for the differential diagnosis between MGUS and multiple myeloma. Am. J. Pathol..

[47] Lacy M.Q., Donovan K.A., Heimbach J.K., Ahmann G.J., Lust J.A. (1999). Comparison of interleukin-1 beta expression by in situ hybridization in monoclonal gammopathy of undetermined significance and multiple myeloma. Blood.

[48] Nakayama-Ichiyama S., Yokote T., Hirata Y., Iwaki K., Akioka T., Miyoshi T., Takayama A., Nishiwaki U., Masuda Y., Nishimura Y. (2012). Immunohistological diagnosis of plasma cell myeloma based on cytoplasmic kappa/lambda ratio of CD138-positive plasma cells. Leuk. Lymphoma.

[49] Pojero F., Flores-Montero J., Sanoja L., Pérez J.J., Puig N., Paiva B., Bottcher S., Van Dongen J.J., Orfao A., EuroFlow group (2016). Utility of CD54, CD229, and CD319 for the identification of plasma cells in patients with clonal plasma cell diseases. Cytom. Part B Clin. Cytom..

[50] Asaoka R., Kiyomatsu H., Miura H., Jono A., Kinoshita T., Takao M., Katagiri T., Oshima Y. (2022). Prognostic potential and pathological validation of a diagnostic application using Raman spectroscopy in the characterization of degenerative changes in the cartilage of the humeral head. J. Biomed. Opt..

